# Low lean mass is associated with lower urinary tract symptoms in US men from the 2005–2006 national health and nutrition examination survey dataset

**DOI:** 10.18632/aging.203480

**Published:** 2021-09-02

**Authors:** Zheng Qin, Junjie Zhao, Jiameng Li, Qinbo Yang, Jiwen Geng, Ruoxi Liao, Baihai Su

**Affiliations:** 1 Department of Nephrology, National Clinical Research Center for Geriatrics, West China Hospital of Sichuan University, Chengdu 610041, China; 2 Med+ Biomaterial Institute of West China Hospital/West China School of Medicine of Sichuan University, Chengdu 610041, China; 3 Med-X Center for Materials, Sichuan University, Chengdu 610041, China; 4 West China School of Medicine, West China Hospital of Sichuan University, Chengdu 610041, China

**Keywords:** low lean mass, lower urinary tract symptoms, NHANES, cross-sectional study

## Abstract

We investigated the relationship between low lean mass (LLM) and lower urinary tract symptoms (LUTS) using the 2005–2006 National Health and Nutrition Examination Survey (NHANES) dataset. We enrolled 959 men with an average age of 52.08 ± 7.91 years and performed weighted multiple regression analysis to determine the independent relationship between exposure variables (LLM, alternate LLM) and outcomes variables (urinary hesitancy, incomplete emptying, urinary frequency, nocturia, daytime LUTS, clinical LUTS) after adjusting for confounding factors. The prevalence of urinary hesitancy (OR = 7.76, *P* < 0.0001), incomplete emptying (OR = 2.49, *P* = 0.0070), urinary frequency (OR = 3.28, *P* < 0.0001), daytime LUTS (OR = 3.88, *P* < 0.0001) and clinical LUTS (OR = 8.11, *P* < 0.0001) was significantly higher among men with LLM compared to men without LLM. Moreover, alternate LLM (ALLM) was positively associated with urinary hesitancy (OR = 17.97, *P* < 0.0001), incomplete emptying (OR = 4.68, *P* = 0.0003), daytime LUTS (OR = 2.47, *P* = 0.0136) and clinical LUTS (OR = 12.18, *P* < 0.0001). These findings demonstrate that both LLM and ALLM were associated with a higher risk of LUTS in men aged ≥ 40 years, which suggested that early management and treatment of lean mass loss may improve or alleviate LUTS.

## INTRODUCTION

Lower urinary tract symptoms (LUTS) such as urinary hesitancy, incomplete emptying, urinary frequency and nocturia remained the most common urologic symptoms that significantly affect the quality of life of more than 90% of men aged 50 *P* < 80 years [1–4]. In the United States, the annual expenditure for LUTS medications is nearly 194 million dollars and represents a significant economic and public health burden [5, 6]. Therefore, there is an urgent need to identify risk factors associated with LUTS.

Low lean mass (LLM) is a key clinical indicator of muscle mass, strength and function [7]. The Foundation for the National Institutes of Health (FNIH) Sarcopenia project data demonstrated that LLM could be used to define sarcopenia, a skeletal muscle disorder that involves age-dependent decline in muscle mass and functional capacity [8]. Several other studies have also shown that LLM could serve as a underlying risk factor for muscle weakness, disability and mortality in the elderly population [9–12].

Although both LLM and LUTS are age-related diseases, the association between them still remains unclear and the knowledge about this topic is limited. Aging induces several changes in body composition, including increased visceral fat and decreased muscle mass. Obesity in the elderly with benign prostatic enlargement could increase the risk of LUTS [13]. Moreover, LLM has been reported to be associated with obesity [14]. Besides, previous studies on community-dwelling adults and older men showed a positive correlation between frailty and LUTS [15, 16]. Huang et al. showed that urinary incontinence was an independent risk factor associated with frailty [17]. Fougere et al. also reported that LLM was partly associated with frailty [18], it can be speculated that there might be a relationship between LLM and LUTS.

Hence, in this study, we explore the association between LLM and LUTS using the 2005–2006 National Health and Nutrition Examination Survey (NHANES) data regarding US men in order to provide more information about LLM and LUTS, which may shed new light on the management and intervention of LUTS at a younger age in clinical practice. We hypothesized that men with LLM were at an increased risk for LUTS.

## RESULTS

### Baseline characteristics of NHANES study participants

**Figure 1:**
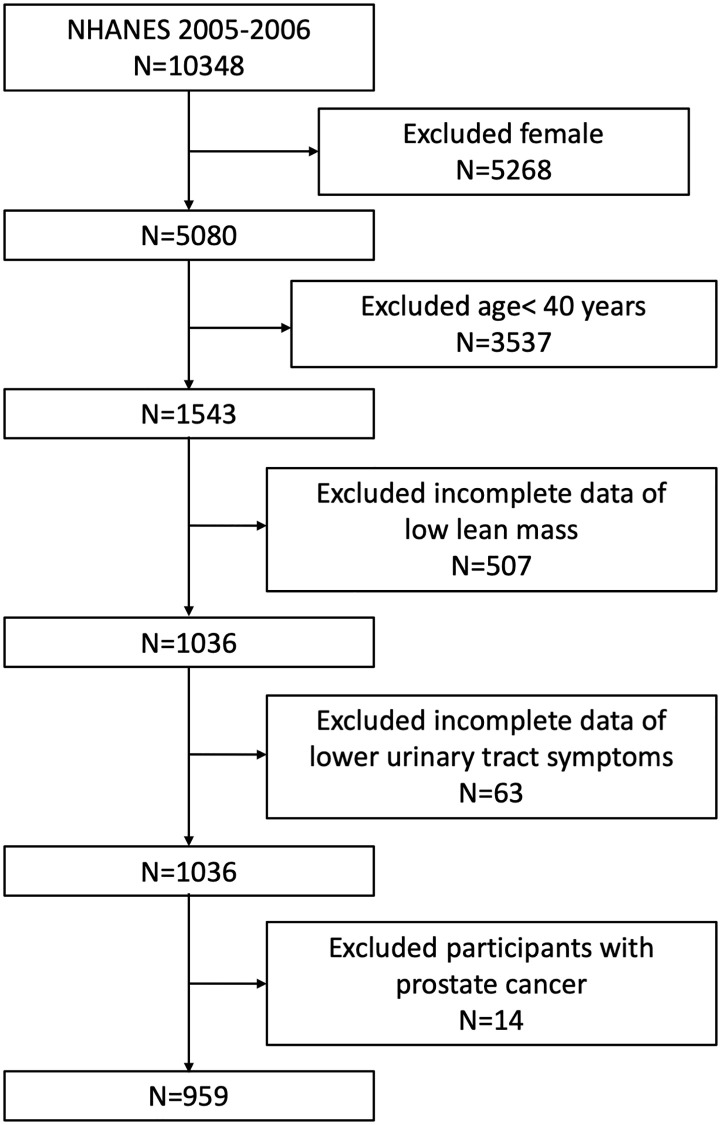
Flowchart of the sample selection from NHANES 2005–2006.

The average age of the 959 men included in our study was 52.08 ± 7.91 years. LLM and alternate LLM (ALLM) was reported by 143 (14.91%) and 55 (5.74%) participants, respectively. Urinary hesitancy, incomplete urine emptying, urinary frequency, and nocturia was reported by 68 (7.10%), 97 (10.11%), 141 (14.70%), and 266 (27.74%) participants, respectively. Daytime LUTS and clinical LUTS (having two or more symptoms above) was reported in 26.38% (*n* = 253) and 13.97% (*n* = 134) of the study participants, respectively (Table 1, Figure 1).

**Table 1:** Baseline characteristics of participants, weighted.

	**Overall**	**Low lean mass**	**Non-low lean mass**	***P* value**
	**(*n* = 959)**	**(*n* = 143)**	**(*n* = 816)**	
Age (years)	52.08 ± 7.91	54.51 ± 8.87	51.72 ± 7.70	0.0003
Race (%)				
Mexican American	6.30	11.77	5.51	0.0001
Other Hispanic	2.43	6.65	1.81	
Non-Hispanic White	77.08	75.10	77.37	
Non-Hispanic Black	9.98	4.30	10.81	
Other Races	4.21	2.18	4.50	
Education level (%)				
Less than high school	5.82	12.11	4.91	0.0043
High school or GED	35.91	37.16	35.73	
Above high school	58.27	50.73	59.37	
RIP (%)				
≤1	8.30	10.21	8.29	0.4840
>1	91.70	89.79	91.71	
Alcohol intake per week (%)				
Never	20.19	24.17	19.62	0.6712
Up to once a week	41.44	42.55	41.28	
2–3 times a week	16.77	15.87	16.90	
4–6 times a week	11.45	8.44	11.88	
Daily or more	10.15	8.97	10.32	
BMI (%)				
Normal weight	20.66	6.13	22.78	<0.0001
Overweight	40.28	25.32	42.46	
Obese	39.06	68.54	34.77	
Insurance (%)	83.45	71.89	85.13	0.0002
Hypertension (%)	38.61	7.30	43.15	<0.0001
Diabetes (%)	12.73	3.48	14.08	0.0011
Congestive heart failure (%)	4.69	0.55	5.29	0.0211
COPD (%)	4.77	0.56	4.93	0.5505
Coronary artery disease (%)	5.43	3.69	6.14	0.0112
Cancer (%)	3.63	2.45	3.80	0.4584
Sleep disorder (%)	9.74	5.49	10.35	0.2012
Smoking status (%)				
Never	39.71	1.88	45.19	<0.0001
Former	33.64	9.74	37.11	
Current	26.65	88.38	17.70	
Comorbidity index (%)				
0	50.96	86.291	45.83	<0.0001
1	34.60	9.99	38.17	
2	9.98	3.11	10.97	
≥3	4.47	0.61	5.03	
Urinary hesitancy (%)	7.26	16.50	5.92	<0.0001
Incomplete emptying (%)	10.28	14.75	9.63	0.0827
Urinary frequency (%)	13.52	18.37	12.82	0.0941
Nocturia (%)	25.33	28.20	24.92	0.4377
Daytime LUTS (%)	25.53	35.44	24.09	0.0073
Clinical LUTS (%)	13.11	27.66	11.00	<0.0001

Abbreviations: BMI: body mass index; GED: general educational development; RIP: ratio of family income to poverty; COPD: chronic obstructive pulmonary disease; LUTS: lower urinary tract symptoms.

### Association between LLM and other clinicopathological characteristics of NHANES study subjects

Table 1 shows details of weighted demographic characteristics and other covariates of the included participants with or without LLM. We observed significantly higher prevalence of older (54.51 ± 8.87 vs. 51.72 ± 7.70 years, *P* = 0.0003), obese (68.54% vs. 34.77%, *P* < 0.0001), and current smokers (88.38% vs. 7.70%, *P* < 0.0001) among men with LLM compared to those without LLM. Furthermore, prevalence of urinary hesitancy (16.50% vs. 5.92%, *P* < 0.0001), daytime LUTS (35.44% vs. 24.09%, *P* = 0.0073), and clinical LUTS (27.66% vs. 11.00%, *P* < 0.0001) was significantly higher in men with LLM compared to those without LLM. The prevalence of incomplete urine emptying, urinary frequency, and nocturia was also higher in participants with LLM compared to those without LLM, but these differences were not statistically significant (all *P* > 0.05; Table 1).

### Association between LUTS and other clinicopathological characteristics of NHANES study subjects

We then compared the demographic characteristics and other covariates between men with or without LUTS such as urinary hesitancy, incomplete emptying, urinary frequency and nocturia (Supplementary Table 1). The prevalence of LLM and ALLM was significantly higher among men with LUTS compared to those without LUTS. The prevalence of hypertension (50.66% vs. 37.67%, *P* = 0.0321), current or former smokers (*P* = 0.0251), LLM (28.79% vs. 11.40%, *P* < 0.0001), and ALLM (25.18% vs. 3.37%, *P* < 0.0001) was significantly higher in study subjects with urinary hesitancy compared to those without urinary hesitancy. The prevalence of ALLM was significant higher in participants with incomplete urine emptying compared to those without incomplete urine emptying (16.93% vs. 3.58%, *P* < 0.0001). The prevalence of LLM was not statistically significant between subjects with or without incomplete urine emptying (18.17% vs. 12.04%, *P* = 0.0827). Moreover, men with urinary frequency and nocturia showed higher prevalence of LLM and ALLM compared to those without urinary frequency and nocturia, but the differences were not statistically significant (all *P* > 0.05).

We further compared demographics of participants with or without daytime LUTS and clinical LUTS. The prevalence of hypertension (54.25% vs. 33.25%, *P* < 0.0001), diabetes (18.44% vs. 10.78%, *P* = 0.0019), sleep disorder (14.36% vs. 8.17%, *P* = 0.0006), current or former smokers (*P* = 0.0472), higher comorbidity index (*P* < 0.0001), LLM (17.58% vs. 10.98%, *P* = 0.0073) and ALLM (8.64% vs. 3.69%, *P* = 0.0020) was significantly higher in men with daytime LUTS than those without daytime LUTS (Supplementary Table 2). Furthermore, prevalence of hypertension (57.91% vs. 35.70%, *P* < 0.0001), diabetes (22.15% vs. 11.31%, *P* = 0.0019), current or former smokers (*P* = 0.0010), higher comorbidity conditions (*P* < 0.0001), LLM (26.72% vs. 10.55%, *P* < 0.0001) and ALLM (16.82% vs. 3.16%, *P* < 0.0001) was significantly higher among men with clinical LUTS compared to those without clinical LUTS (Supplementary Table 3).

### Association between LLM and LUTS

We performed weighted multiple regression analysis to determine the association between LLM and ALLM with LUTS. The estimated odd ratios (ORs) for the relationship between LLM and LUTS are shown in Table 2 and those for the association between ALLM and LUTS are shown in Table 3.

**Table 2:** Association between low lean mass and lower urinary tract symptoms, weighted.

	**OR (95% CI), *P* value**	
	**Non-adjusted (*n* = 959)**	**Adjusted (*n* = 877)**
Urinary hesitancy		
No	Reference	Reference
Yes	3.04 (1.77, 5.24) <0.0001	7.76 (3.58, 16.82) <0.0001
Incomplete emptying		
No	Reference	Reference
Yes	1.34 (0.78, 2.32) 0.2892	2.49 (1.28, 4.85) 0.0070
Urinary frequency		
No	Reference	Reference
Yes	1.35 (0.85, 2.16) 0.2042	3.28 (1.81, 5.93) <0.0001
Nocturia		
No	Reference	Reference
Yes	1.10 (0.74, 1.63) 0.6363	1.40 (0.86, 2.29) 0.1755
Daytime LUTS		
No	Reference	Reference
Yes	1.56 (1.07, 2.29) 0.0211	3.88 (2.37, 6.35) <0.0001
Clinical LUTS		
No	Reference	Reference
Yes	2.35 (1.52, 3.63) 0.0001	8.11 (4.26, 15.46) <0.0001

Abbreviations: LUTS: lower urinary tract symptoms; OR: odd ratio.

Adjusted for age, BMI, race, education level, income, insurance, alcohol intake, diabetes, hypertension, smoking status, comorbidity index.

**Table 3:** Association between alternate low lean mass and lower urinary tract symptoms, weighted.

	**OR (95% CI), *P* value**	
	**Non-adjusted (*n* = 959)**	**Adjusted (*n* = 877)**
Urinary hesitancy		
No	Reference	Reference
Yes	6.18 (3.21, 11.93) <0.0001	17.97 (6.88, 46.90) <0.0001
Incomplete emptying		
No	Reference	Reference
Yes	3.47 (1.81, 6.63) 0.0002	4.68 (2.04, 10.70) 0.0003
Urinary frequency		
No	Reference	Reference
Yes	1.34 (0.66, 2.73) 0.4166	1.70 (0.69, 4.21) 0.2486
Nocturia		
No	Reference	Reference
Yes	1.10 (0.60, 2.01) 0.7492	1.16 (0.54, 2.51) 0.6975
Daytime LUTS		
No	Reference	Reference
Yes	1.56 (0.87, 2.77) 0.1335	2.47 (1.20, 5.08) 0.0136
Clinical LUTS		
No	Reference	Reference
Yes	3.73 (2.06, 6.74) <0.0001	12.18 (5.26, 28.22) <0.0001

Abbreviations: LUTS: lower urinary tract symptoms; OR: odd ratio.

Adjusted for age, BMI, race, education level, income, insurance, alcohol intake, diabetes, hypertension, smoking status, comorbidity index.

In the non-adjusted model, urinary hesitancy (OR = 3.04, 95% CI: 1.77–5.24, *P* < 0.0001), daytime LUTS (OR = 1.56, 95% CI: 1.07–2.29, *P* = 0.0211) and clinical LUTS (OR = 2.35, 95% CI: 1.52–3.63, *P* = 0.0001) were significantly associated with LLM. However, the association between LLM and LUTS parameters such as incomplete urine emptying, urinary frequency, and nocturia was not statistically significant (all *P* > 0.05). In the adjusted model, LLM was associated with significantly higher risk of urinary hesitancy (OR = 7.76, 95% CI: 3.58–16.82, *P* < 0.0001), incomplete urine emptying (OR = 2.49, 95% CI: 1.28–4.85, *P* = 0.0070), urinary frequency (OR = 3.28, 95% CI: 1.81–5.93, *P* < 0.0001), daytime LUTS (OR = 3.88, 95% CI: 2.37 = 6.35, *P* < 0.0001) and clinical LUTS (OR = 8.11, 95% CI: 4.26–15.46, *P* < 0.0001), but was not associated with nocturia (OR = 1.40, 95% CI: 0.86–2.29, *P* = 0.1755).

Furthermore, adjusted model results showed that ALLM was significantly associated with urinary hesitancy (OR = 17.97, 95% CI: 6.88–46.90, *P* < 0.0001), incomplete urine emptying (OR = 4.68, 95% CI: 2.04–10.70, *P* = 0.0003), daytime LUTS (OR = 2.47, 95% CI: 1.20–5.08, *P* = 0.0136), and clinical LUTS (OR = 12.18, 95% CI: 5.26–28.22, *P* < 0.0001), but was not associated with urinary frequency and nocturia (all *P* > 0.05).

In addition, we observed significant differences in results from the non-adjusted and adjusted models for the association between LLM and LUTS parameters such as incomplete urine emptying (non-adjusted, OR = 1.34, 95% CI: 0.78–2.32; adjusted, OR = 2.49, 95% CI: 1.28–4.85), urinary frequency (non-adjusted, OR = 1.35, 95% CI: 0.85–2.16; adjusted, OR = 3.28, 95% CI: 1.81–5.93) for LLM, and the association between ALLM and LUTS (non-adjusted, OR = 1.56, 95% CI: 0.87–2.77; adjusted, OR = 2.47, 95% CI: 1.20–5.08). These differences may be due to confounding bias between lean mass loss and LUTS in the non-adjusted model, but they were eliminated in the adjusted model. Therefore, we postulate that adjusted OR is of greater clinical significance.

## DISCUSSION

In this cross-sectional study included 959 men aged ≥ 40 years old, we investigated the relationship between LLM and LUTS using weighted multiple regression analysis based on the NHANES dataset (2005–2006). After adjusting for covariates such as age, BMI, race, education level, income, insurance, alcohol intake, diabetes, hypertension, smoking status and comorbidity index, the risk of urinary hesitancy, incomplete urine emptying, urinary frequency, daytime LUTS, and clinical LUTS was significantly higher among men with LLM compared to those without LLM. Moreover, ALLM was associated with urinary hesitancy, incomplete urine emptying, daytime LUTS and clinical LUTS. Since both LLM and ALLM showed positive association with LUTS, we postulate that treatment and management of lean mass loss at a younger age might be beneficial to improve or alleviate LUTS.

To the best of our knowledge, this is the first study to evaluate the association between LLM and LUTS in older US men. Previous studies have reported association of LUTS with several other clinicopathological factors. Gacci et al. reported that central obesity was a risk factor for LUTS progression after prostatic surgery to resolve benign prostatic enlargement or prostate cancer [19]. A cross-sectional study by Soma et al. including 710 community-dwelling Japanese participants aged ≥ 60 years old demonstrated that subjects with LUTS were potentially frailer than those without LUTS, indicating a positive correlation between frailty and LUTS [15]. Huang et al. reported that urinary incontinence was independently associated with frailty among older adults living in Taiwanese rural communities [17]. Bauer et al. also observed that frailty was common among older men with LUTS and should be considered during the initial urological evaluation [16]. Both obesity and frailty were associated with LLM [14, 18], thereby suggesting a possible relationship between LLM and LUTS. Our results demonstrated that men with LLM or ALLM were associated with a higher risk for LUTS than men without LLM. Pacini et al. investigated the association between LUTS and clinicopathological parameters in systemic sclerosis patients, and demonstrated a significant positive correlation between overactive bladder and sarcopenia, which was consistent with our findings [20].

LLM and LUTS share several common risk factors. Many studies have shown that both LLM and LUTS are highly prevalent in aged individuals [2, 8, 21]. Moreover, a cross-sectional study by Fantus et al. reported that sleep disorder was associated with the occurrence of nocturia and daytime LUTS [22]. Furthermore, accumulated evidences have also proven that reductions in the duration and quality of sleep and increases in the prevalence of circadian rhythm and sleep disorder with age enhanced the risk of LLM by promoting proteolysis, modifying the body composition, and increasing the risk of insulin resistance [23]. These findings demonstrated that sleep disorders may also act as a common risk factor of LLM and LUTS. Thus, we designated sleep disorder as a covariate in the adjusted weighted multiple regression models to obtain more reliable results.

The exact mechanisms underlying the association between LLM and LUTS are not clear. Both LLM and LUTS are closely related to hormonal deregulation. Hypogonadism is commonly observed in subjects with LLM, persistent hypogonadism promotes prostate enlargement and worsens LUTS through a hypogonadism-obesity-benign prostatic hyperplasia-LUTS model [24, 25]. In our study, the prevalence of obesity was significantly higher in men with LUTS compared to non-obese individuals (68.54% vs. 34.11%, *P* < 0.0001), which was consistent with previous studies. Moreover, lean mass loss was associated with deficiencies in growth hormone (GH) and testosterone [9, 26]. Patients with adult growth hormone deficiency (AGHD) showed reduced lean body mass and muscle strength compared to than healthy individuals [27]. In addition, AGHD patients who received GH treatment showed a lower prevalence of LLM compared to those that did not receive GH treatment [28]. Several studies also have demonstrated that declining testosterone levels are associated with LUTS. Kim et al. reported that testosterone levels were significantly reduced in patients with severe nocturia [29]. Rabijewski et al. studied prevalence of erectile dysfunction and LUTS in middle-aged and elderly men with pre-diabetes, and reported a positive association between lower testosterone levels with severe LUTS [30]. Wu et al. reported higher prevalence of nocturia in men with reduced testosterone levels who underwent transurethral prostate resection as well [31]. Decreased testosterone levels may contribute to LUTS by inducing hyperactivity of the autonomic nervous system. Testosterone could play a critical role in the reflex activity of the pelvic autonomic nervous system by interacting with the postsynaptic non-genomic receptors located in the epithelium of the urethra and bladder, thereby increasing bladder volume and compliance, and by reducing detrusor pressure at male maximum flow to inhibit detrusor activity [32, 33]. Both testosterone and GH regulated the skeletal muscle protein synthesis and degradation through several common signaling pathways [34]. GH enhances the metabolic effects of testosterone, which in turn affects the neuroendocrine rhythm of GH [35]. The secretion of these two hormones is positively related and interplayed closely. Hence, we postulate a relationship between LLM and LUTS. However, further studies are necessary to understand the potential mechanisms regulating this association between LLM and LUTS.

Our study has several strengths. Firstly, our study was based on the data from NHANES, which was a nationwide, population-based sampling data obtained using a standard protocol for NHANES project. Moreover, the study was more representative because the analysis was performed by considering an appropriate NHANES sampling weight and involved a complex multistage cluster survey design. Secondly, we adjusted for confounding covariates to ensure our results reliable and could be applied to a wider range of individuals. We also selected covariates mainly based on previous studies in order to assess the relationship of LUTS with other exposure variables. However, there are several limitations in this study. Due to the cross-sectional study design, we could not obtain the casual relationship between LLM and LUTS. Besides, LUTS was based on self-reported symptoms by the study subjects. This may have resulted in recall bias and might impact the accuracy of our results. Moreover, this study was based on only one NHANES cycle (2005 to 2006) because only this cycle included data regarding both on appendicular lean mass and urinary symptoms. Therefore, the sample capacity of our study was relatively small. Moreover, the data of some potential confounders such as the use of diuretics, alpha blockers, surgery for benign prostate hyperplasia and some other medications, was not available in the NHANES data. We cannot completely rule out residual confounders due to unmeasured or unknown covariates. Therefore, the influence of these confounders on the association between LLM and LUTS is not known. Finally, the data regarding muscle strength was not available in NHANES to assess LLM.

In conclusion, our study demonstrated that both LLM and ALLM were associated with higher risk of LUTS in men aged ≥ 40 years old. Therefore, we postulate that management and treatment of LLM and ALLM at a younger age might be beneficial to alleviate LUTS. However, further studies are still needed to validate our findings.

## METHODS

### Data source

We obtained publicly available data from the National Health and Nutrition Examination Survey (NHANES), a cross-sectional study to assess the nutrition and health status of adults and children in the United States [36]. NHANES is conducted on a repeated two-year cycle by the National Center for Health Statistics (NCHS). All NHANES data are publicly available at https://www.cdc.gov/nchs/nhanes/.

Our study was based on the data from NHANES 2005 to 2006, since only this cycle contained information on both appendicular lean mass and questionnaires related to urinary symptoms. The NCHS Ethics Review Board granted the approval for NHANES to conduct human subject study. Written informed consent was obtained from all each participant.

### Study population

Men aged ≥40 years who both answered questionnaires on LUTS and underwent body composition measurements were enrolled in our study.

Out of the originally enrolled 10348 individuals in the NHANES 2005–2006, we excluded females (*n* = 5268), individuals aged below 40 years old (*n* = 3537), individuals missing the examination data relating to LLM (*n* = 507), individuals missing LUTS questionnaires data (*n* = 63) and participants with prostate cancer (*n* = 14). Therefore, our final analysis included 959 men aged ≥ 40 years (Figure 1).

### Body composition measurements

Body composition measurements were conducted by well-trained technicians using dual energy X-ray absorptiometry (DXA) QDR-4500 Hologic Scanner (Bedford, MA). DXA scans were not performed for participants with a self-reported use of radiographic contrast material (barium) in the seven days before scans, weight above 450 pounds and height above 192.5 cm. The data on total lean mass, appendicular lean mass (ALM), bone mineral content, bone area and fat mass were reported and available in NHANES 2005–2006. Although total lean mass was available, we could not find a proper cut-off value to define low lean mass according to previous studies regarding total lean mass data. Therefore, we used ALM to define LLM and ALLM in our analysis. ALM was defined as the sum of muscle mass of all four upper and lower extremity limbs. In our analysis, we define ALM adjusted for BMI (ALM_BMI_) and ALM to classify low lean mass according to FNIH in 2014 [37]. For men, low lean mass (LLM) was defined as ALM_BMI_< 0.789 and alternate low lean mass (ALLM) was defined as ALM <19.75 kg. Low lean mass and alternate low lean mass were used as exposure variables.

### Lower urinary tract symptoms

LUTS were assessed based on answers to the following four questions, including: (1)“Do you usually have trouble starting to urinate?” for urinary hesitancy; (2)“After urinating, does your bladder feel empty?” for incomplete urine emptying; (3) “How often do you have urinary leakage?” for urinary frequency; and (4) “During the past 30 days, how many times per night did you most typically get up to urinate, from the time you went to bed at night until the time you got up in the morning?” for nocturia.

Nocturia was defined as waking up at least twice per night to urinate during sleep. If a participant answered yes to urinary hesitancy, or incomplete emptying, or at least 1 to urinary frequency, he was considered to have a daytime LUTS. An individual with 2 or more the mentioned symptoms above (urinary hesitancy, incomplete emptying, urinary frequency and nocturia) was considered to have clinical LUTS. Urinary hesitancy, incomplete emptying, urinary frequency, nocturia, daytime LUTS and clinical LUTS were used as outcome variables.

### Covariates

The selection of covariates was mainly based on previous studies that assessed the relationship of LUTS with other exposure variables [22, 38]. We included data regarding demographic (age, race), socioeconomic conditions (education level, ratio of family income to poverty, insurance), lifestyle (alcohol intake, smoking status) and health status (body mass index, sleep disorder, diabetes, other comorbidities) as covariates.

Comorbidity index reflected the health status of patients and was of great significance for disease management, clinical treatment and prognosis [39]. Comorbid conditions included diabetes mellitus, congestive heart failure, chronic obstructive pulmonary disease (emphysema and/or chronic bronchitis), coronary artery disease, cancer and hypertension. The data about comorbid conditions was obtained from answers to the questionnaire in NHANES by the study subjects. An ordinal comorbidity index was then estimated based on the total number of combined condition reported by the study subjects as reported previously [22]. Hypertension was defined based on self-reported hypertension diagnosis, or a blood pressure measurement ≥140/90 mmHg [40]. Diabetes was defined as self-reported diabetes diagnosis, use of oral hypoglycemic agents or insulin, a fasting glucose level ≥ 126 mg/dL, or a plasma glucose level ≥ 200 mg/dL at 2 h after oral glucose tolerance test [41]. Congestive heart failure, chronic obstructive pulmonary disease, coronary artery disease and cancer were all based on a self-reported answer regarding previous diagnosis.

Categorical variables included race (Mexican American, other Hispanic, non-Hispanic White, non-Hispanic Black and other races), education level (less than high school, high school or general educational development and above high school), ratio of family income to poverty (≤1 and >1), whether have insurance (yes or not), alcohol intake (never, up to once a week, 2–3 times a week, 4–6 times a week and daily or more), body mass index (BMI in Kg/m^2^; categorized as <25 for normal weight, 25–29.9 as overweight and ≥30 as obese), diabetes (yes or not), smoking status (never, former or current). All detailed measurement process of these variable was publicly available at https://www.cdc.gov/nchs/nhanes/.

### Statistical analysis

Statistical analysis was conducted according to CDC guidelines using Empower software (https://www.empowerstats.com/; X&Y solutions, Inc., Boston MA) and R version 3.4.3 (https://www.R-project.org, The R Foundation) [42]. All statistical analysis was performed by considering appropriate NHANES sampling weights using the complex multistage cluster survey design. Continuous variables were represented as mean with standard deviation, whereas, categorical variables were presented as frequency or percentage. Weighted student’s *t* test (for continuous variables) or weighted chi-square test (for categorical variables) were used to assess the differences between groups classified according to LLM and LUTS status. Weighted multiple regression analysis was performed to estimate the independent relationship between exposure variables (LLM, ALLM) and outcomes variables (urinary hesitancy, incomplete emptying, urinary frequency, nocturia, daytime LUTS, clinical LUTS) using non-adjusted model and model adjusted for age, BMI, race, education level, income, insurance, alcohol intake, diabetes, hypertension, smoking status and comorbidity index. *P* < 0.05 was considered statistically significant.

## Supplementary Materials

Supplementary Tables
