# Supplementary material for: Low lean mass is associated with lower urinary tract symptoms in US men from the 2005–2006 national health and nutrition examination survey dataset
Source: Aging (Albany NY). 2021 Sep 2;13(17):21421–34. doi: 10.18632/aging.203480 (PMC8457570; doi:10.18632/aging.203480)
Supplement: Supplementary Tables [file aging-13-203480-s001.pdf]

SUPPLEMENTARY TABLES

Supplementary Table 1. Participants demographics by lower urinary tract symptoms, weighted.

	Overall	Urinary hesitancy			Incomplete emptying			Urinary frequency			Nocturia			
	(*n* = 959)	Yes (*n* = 68)	No (*n* = 891)	*P* value	Yes (*n* = 97)	No (*n* = 862)	*P* value	Yes (*n* = 141)	No (*n* = 818)	*P* value	Yes (*n* = 266)	No (*n* = 593)	*P* value	
Age (years)	52.08 ± 7.91	51.91 ± 8.27	52.09 ± 7.88	0.8553	51.61 ± 8.47	52.13 ± 7.84	0.5358	52.39 ± 8.23	52.03 ± 7.86	0.6343	52.02 ± 7.68	52.26 ± 8.55	0.6808	
Race (%)														
Mexican American	6.30	5.51	6.36	0.9353	4.39	6.52	0.8920	7.30	6.14	0.4107	7.64	5.84	0.6698	
Other Hispanic	2.43	3.64	2.33		1.66	2.51		2.54	2.41		2.43	2.43		
Non-Hispanic White	77.08	76.47	77.13		80.43	76.70		73.10	77.71		73.72	78.23		
Non-Hispanic Black	9.98	11.19	9.89		9.72	10.01		14.24	9.32		11.51	9.47		
Other Races	4.21	3.19	4.28		3.80	4.25		2.82	4.42		4.70	4.04		
Education level (%)														
Less than high school	5.82	3.94	5.97	0.7358	7.39	5.64	0.3024	4.64	6.00	0.7658	5.82	5.82	0.9920	
High school or GED	35.91	38.60	35.70		41.50	35.27		37.87	35.60		35.58	36.02		
Above high school	58.27	57.46	58.34		51.11	59.09		57.49	58.40		58.60	58.16		
RIP (%)														
≤ 1	8.30	8.05	9.99	0.4686	8.01	10.82	0.3375	8.29	8.31	0.9950	7.60	10.35	0.1797	
> 1	91.70	91.95	90.01		91.99	89.18		91.71	91.69		92.40	89.65		
Alcohol intake per week (%)														
Never	20.19	18.55	20.31	0.9849	16.83	20.56	0.3200	18.79	20.41	0.8778	17.35	21.12	0.2336	
Up to once a week	41.44	42.77	41.34		37.68	41.85		43.59	41.10		37.91	42.60		
2–3 times a week	16.77	16.51	16.79		20.54	16.36		18.31	16.53		19.30	15.94		
4–6 times a week	11.45	10.39	11.53		16.80	10.86		11.38	11.46		14.18	10.56		
Daily or more	10.15	11.79	10.03		8.14	10.37		7.93	10.49		11.26	9.78		
BMI (%)														
Normal weight	20.66	22.26	20.54	0.8933	21.79	20.53	0.9005	18.40	21.02	0.0528	21.68	20.32	0.6666	
Overweight	40.28	37.77	40.47		41.19	40.17		49.88	38.77		41.69	39.80		
Obese	39.06	39.97	38.99		37.01	39.30		31.71	40.22		36.63	39.89		
Insurance (%)	83.45	84.33	83.38	0.8366	83.44	83.45	0.9987	88.73	82.62	0.0820	86.15	82.53	0.1902	
Hypertension (%)	38.61	50.66	37.67	0.0321	42.39	38.18	0.4163	58.42	35.51	<0.0001	53.09	33.70	<0.0001	
Diabetes (%)	12.73	16.56	12.43	0.3200	15.91	12.37	0.3177	22.14	11.26	0.0006	21.58	9.73	<0.0001	
CHF (%)	4.69	4.15	4.73	0.8274	5.77	4.56	0.5902	10.69	3.75	0.0005	10.55	2.69	<0.0001	
COPD (%)	4.77	4.97	4.75	0.9351	4.44	7.64	0.1576	12.15	3.62	<0.0001	5.30	4.59	0.6510	
CAD (%)	5.43	8.57	5.18	0.2304	5.23	7.20	0.4115	5.50	5.42	0.9714	10.25	3.79	0.0001	
Cancer (%)	3.63	7.22	3.35	0.0961	7.41	3.20	0.0342	3.94	3.58	0.8368	5.74	2.91	0.0415	
Sleep disorder (%)	9.74	4.29	10.16	0.2559	3.36	10.47	0.0682	16.27	8.72	0.0227	14.36	8.17	0.0006	
Smoking status (%)														
Never	39.71	27.69	40.65	0.0251	29.78	40.85	0.0831	36.39	40.23	0.5821	34.20	41.58	0.1121	
Former	33.64	47.85	32.53		36.71	33.29		33.52	33.66		37.78	32.24		
Current	26.65	24.45	26.82		33.51	25.86		30.09	26.11		28.02	26.18		
Comorbidity index (%)														
0	50.96	40.61	51.77	0.0833	44.24	51.72	0.2172	29.45	54.32	<0.0001	50.96	35.86	56.08	<0.0001
1	34.60	36.76	34.43		35.92	34.45		44.61	33.03		34.60	37.08	33.76	
2	9.98	18.12	9.34		11.86	9.76		15.70	9.08		9.98	18.25	7.17	
≥3	4.47	4.51	4.47		7.98	4.07		10.24	3.57		4.47	8.82	2.99	
LLM (%)	12.67	28.79	11.40	<0.0001	18.17	12.04	0.0827	17.21	11.96	0.0941	14.10	12.18	0.4377	0.4377
ALLM (%)	4.95	25.18	3.37	<0.0001	16.93	3.58	<0.0001	6.62	4.69	0.3459	6.94	4.27	0.0980	0.0980

Abbreviations: BMI: body mass index; GED: general educational development; RIP: ratio of family income to poverty; CHF: congestive heart failure; COPD: chronic obstructive pulmonary disease; CAD: Coronary artery disease; LLM: low lean mass; ALLM: alternate low lean mass.

Supplementary Table 2. Participants demographics by daytime LUTS, weighted.

	Overall	Daytime LUTS	Non-Daytime LUTS	***P* value**
	**(*n* = 959)**	**(*n* = 253)**	**(*n* = 706)**	
Age (years)	52.08 ± 7.91	51.72 ± 8.14	52.20 ± 7.83	0.4084
Race (%)				
Mexican American	6.30	6.64	6.18	0.7429
Other Hispanic	2.43	3.04	2.21	
Non-Hispanic White	77.08	74.61	77.93	
Non-Hispanic Black	9.98	11.78	9.37	
Other Races	4.21	3.93	4.30	
Education level (%)				
Less than high school	5.82	5.40	5.96	0.0962
High school or GED	35.91	41.63	33.95	
Above high school	58.27	52.97	60.09	
RIP (%)				
≤ 1	8.30	7.86	9.57	0.4026
> 1	91.70	92.14	90.43	
Alcohol intake per week (%)				
Never	20.19	20.23	20.18	0.8819
Up to once a week	41.44	41.60	41.39	
2–3 times a week	16.77	18.09	16.33	
4–6 times a week	11.45	11.60	11.40	
Daily or more	10.15	8.48	10.71	
BMI (%)				
Normal weight	20.66	20.76	20.63	0.0569
Overweight	40.28	46.10	38.27	
Obese	39.06	33.14	41.10	
Insurance (%)	83.45	12.40	17.97	0.0430
Hypertension (%)	38.61	54.25	33.25	<0.0001
Diabetes (%)	12.73	18.44	10.78	0.0019
CHF (%)	4.69	7.54	3.71	0.0142
COPD (%)	4.77	9.96	2.99	<0.0001
CAD (%)	5.43	6.81	4.96	0.2705
Cancer (%)	3.63	6.05	2.80	0.0188
Sleep disorder (%)	9.74	14.36	8.17	0.0006
Smoking state (%)				
Never	39.71	33.38	41.88	0.0472
Former	33.64	38.82	31.87	
Current	26.65	27.80	26.25	
Comorbidity index (%)				
0	50.96	34.28	56.67	<0.0001
1	34.60	42.45	31.91	
2	9.98	14.98	8.26	
≥3	4.47	8.30	3.16	
LLM (%)	12.67	17.58	10.98	0.0073
ALLM (%)	4.95	8.64	3.69	0.0020

Abbreviations: BMI: body mass index; RIP: ratio of family income to poverty; GED: general educational development; CHF: congestive heart failure; COPD: chronic obstructive pulmonary disease; CAD: Coronary artery disease; LLM: low lean mass; ALLM: alternate low lean mass.

Supplementary Table 3. Participants demographics by clinical LUTS, weighted.

	Overall (*n* = 959)	Clinical LUTS (*n* = 134)	Non-Clinical LUTS (*n* = 825)	*P* value
Age (years)	52.08 ± 7.91	51.59 ± 8.28	52.15 ± 7.85	0.4611
Race (%)				
Mexican American	6.30	5.72	6.39	0.3210
Other Hispanic	2.43	3.74	2.23	
Non-Hispanic White	77.08	75.17	77.37	
Non-Hispanic Black	9.98	13.44	9.46	
Other Races	4.21	1.93	4.55	
Education level (%)				
Less than high school	5.82	4.72	5.98	0.4817
High school or GED	35.91	40.52	35.21	
Above high school	58.27	54.76	58.80	
RIP (%)				
≤ 1	8.30	7.89	11.02	0.2352
> 1	91.70	92.11	88.98	
Alcohol intake per week (%)				
Never	20.19	15.73	20.84	0.5387
Up to once a week	41.44	39.47	41.73	
2–3 times a week	16.77	19.94	16.31	
4–6 times a week	11.45	14.12	11.06	
Daily or more	10.15	10.74	10.06	
BMI (%)				
Normal weight	20.66	18.30	21.02	0.7717
Overweight	40.28	40.90	40.18	
Obese	39.06	40.80	38.80	
Insurance (%)	83.45	87.06	82.90	0.2423
Hypertension (%)	38.61	57.91	35.70	<0.0001
Diabetes (%)	12.73	22.15	11.31	0.0019
CHF (%)	4.69	9.65	3.94	0.0048
COPD (%)	4.77	8.77	4.16	0.0238
CAD (%)	5.43	9.19	4.86	0.0457
Cancer (%)	3.63	8.81	2.85	0.0009
Sleep disorder (%)	9.74	10.61	9.61	0.7962
Smoking state (%)				
Never	39.71	24.59	41.99	0.0010
Former	33.64	41.52	32.45	
Current	26.65	33.89	25.56	
Comorbidity index (%)				
0	50.96	31.29	53.92	<0.0001
1	34.60	40.05	33.78	
2	9.98	19.08	8.60	
≥3	4.47	9.59	3.70	
LLM (%)	12.67	26.72	10.55	<0.0001
ALLM (%)	4.95	16.82	3.16	<0.0001

Abbreviations: BMI: body mass index; GED: general educational development; RIP: ratio of family income to poverty; CHF: congestive heart failure; COPD: chronic obstructive pulmonary disease; CAD: Coronary artery disease; LLM: low lean mass; ALLM: alternate low lean mass.
